# The Mitochondrial Genome of *Arctica islandica*; Phylogeny and Variation

**DOI:** 10.1371/journal.pone.0082857

**Published:** 2013-12-02

**Authors:** Gernot Glöckner, Ivonne Heinze, Matthias Platzer, Christoph Held, Doris Abele

**Affiliations:** 1 Institute for Biochemistry I, Medical Faculty, University of Cologne, Köln and Leibniz-Institute of Freshwater Ecology and Inland Fisheries, IGB, Berlin, Germany; 2 Leibniz Institute for Age Research, Fritz-Lipmann Institute, Jena, Germany; 3 Alfred Wegener Institute for Polar and Marine Research, AWI, Bremerhaven, Germany; University of Florence, Italy

## Abstract

*Arctica islandica* is known as the longest-lived non-colonial metazoan species on earth and is therefore increasingly being investigated as a new model in aging research. As the mitochondrial genome is associated with the process of aging in many species and bivalves are known to possess a peculiar mechanism of mitochondrial genome inheritance including doubly uniparental inheritance (DUI), we aimed to assess the genomic variability of the *A. islandica* mitochondrial DNA (mtDNA). We sequenced the complete mitochondrial genomes of *A. islandica* specimens from three different sites in the Western Palaearctic (Iceland, North Sea, Baltic Sea). We found the *A. islandica* mtDNA to fall within the normal size range (18 kb) and exhibit similar coding capacity as other animal mtDNAs. The concatenated protein sequences of all currently known Veneroidea mtDNAs were used to robustly place *A. islandica* in a phylogenetic framework. Analysis of the observed single nucleotide polymorphism (SNP) patterns on further specimen revealed two prevailing haplotypes. Populations in the Baltic and the North Sea are very homogenous, whereas the Icelandic population, from which exceptionally old individuals have been collected, is the most diverse one. Homogeneity in Baltic and North Sea populations point to either stronger environmental constraints or more recent colonization of the habitat. Our analysis lays the foundation for further studies on *A. islandica* population structures, age research with this organism, and for phylogenetic studies. Accessions for the mitochondrial genome sequences: KC197241 Iceland; KF363951 Baltic Sea; KF363952 North Sea; KF465708 to KF465758 individual amplified regions from different speciemen

## Introduction

The Iceland clam *Arctica islandica* (Linnaeus 1767, common name „ocean quahog”) is the longest-lived non-colonial metazoan with a recorded maximum lifespan potential (MLSP) of more than 507 years reported for the subpolar population from the North Icelandic shelf [1]. Because of its outstanding longevity, *A. islandica* has garnered interest as a new invertebrate model of aging and minimal senescence [2]. Individual shells of this interminably growing species can reach large sizes (approaching 100mm shell length) and can also become very heavy as layers of aragonite are added to the inner shell wall. Because of its annual growth increment formation, *A. islandica* has been used successfully as a high-resolution marine environmental archive [3]. The species has a wide geographical distribution in the Northern hemisphere covering temperate to subarctic regions in eastern (European) and western (American) North Atlantic shelf regions. In the eastern Atlantic, stable populations exhibiting the full lifespan occur around Iceland, in the Irish Sea, and on the entire Scandinavian Atlantic shelf. In these fully marine environments, *Arctica* forms dense stocks between 30 and 60m water depth [4]. The German Bight and southern North Sea stocks have thinned dramatically during the past 50 years, owing mostly to the impact of intense beam trawl fishery [5]. Nearly no young premature animals can be found here and MLSP is limited to <100y [5,6]. Marginal but stable populations further exist in brackish White (WS) and Baltic (BS) seas. Low and variable salinity correlate with a dramatically decreased population-specific lifespan in the White and Baltic Sea [7]. First investigations of the transcriptome of this longevity champion indicate that specimens from low salinity environments (Baltic Sea) transcribe a number of stress genes upon exposure to anoxia, whereas individuals from Iceland and the North Sea largely shut down gene transcription under anoxia and, instead, enter a state of metabolic rate reduction in which only cellular maintenance is supported [8]. The mitochondria, their chemical composition, their energy conserving electron transport systems and their capacity to generate free radicals under different metabolic and stress conditions are being extensively studied and related to longevity of vertebrate and invertebrate species [9-14]. 

Bivalves are grouped into five subclasses according to a molecular phylogeny [15]. *A. islandica* belongs to the Heterodonta, order Veneroida, family Arcticidae. Currently 20 complete mitochondrial genomes from this subclass are available in public databases, enabling a thorough gene order comparison between them. Metazoan mitochondria have a size range of 11 to 32 kb, the latter referring to the scallop *Placopecten magellanicus* (NC_007234). They normally encode 13 protein coding genes and up to 22 tRNA genes together with the two mitochondrial rRNA species [16]. The number of tRNA genes is the least conserved feature in the mtDNA and can vary between 2 to nearly 30 tRNA sequences in metazoan mitochondrial genomes [16]. Cnidaria, some Porifera, and arrow worms are known to have severely reduced set of mitochondrially encoded tRNAs, but this condition is expected to have evolved independently many times throughout the phylogenetic tree [17,18]. Further, molluscan mtDNA exhibit the highest variability observed in terms of genome size, mainly due to segmental duplications with rearrangements.

Our study aiming at the deciphering and analysis of the *A. islandica* mitochondrial genome was motivated by three objectives: i) to establish a data set for the correct placement of *A. islandica* in the phylogenetic context ii) to assess its overall mt genome variability and iii) test haplotype diversity in a set of specimen. 

## Results and Discussion

### Sampling

The three selected habitats differ considerably in their salinity and mean temperature regimes. While the salinity in the North sea (33-34‰) is like that of the Icelandic shelf (34‰), the salinity at the Baltic sea sampling site is much lower with only 25 ‰ but fluctuations are high (between 14 and 28‰), depending on episodic inflow of more saline water via the Kattegat. The mean annual water temperature around Iceland is 5°C, highest summer temperatures approach 10°C, while the Baltic and North Sea sampling sites approach a mean annual water temperature of 10 °C. 

The Iceland population has a maximum reported lifespan of 507 years [1] and specimens older than 150 years have repeatedly been found [19]. Here, the animals live between 10 and 40 m water depth. Animals from the North Sea around Helgoland have a maximum observed lifespan of 100 years and the shell length approaches 100mm. Mean shell length in the population is 85mm. Baltic Sea population maximum lifespan is 30 years and maximum shell length is 50 mm. Despite the short lifespan this brakish population is comparably dense with many new recruits.

In total, we sampled 17 specimens (five from the Baltic Sea, and six from Iceland and North Sea, respectively, Table 1). 

**Table 1:** Sampled specimens of *A. islandica*.

ID	date of catch	region	coordinates
276614 276617 27661 27663 27668	27.06.2006	Baltic Sea, Kiel Bay	N 54°32.6´- E 10°42.1´
101ISMRD 108MRD10IS 111MRD10IS 72IS10N 86IS10N 95MRD10IS	15.08.2008	Iceland, NE	N 66° 01.44´ - W 14° 50.91´
10MRD 24N10 33MRD10 37N10 44N10 47MRD10	20.05.2008	North Sea, Helgoland	N 54°10.44´- E 007° 48.12´

### Mitochondrial genome properties

Sequencing of one randomly selected individual from each sampling site/region resulted in a consensus mitochondrial sequence of 18294 bases after gap closure procedures (see Methods). In total, we obtained a consensus sequence with an average coverage of more than 40 over the whole genome (Table 2). We used this consensus sequence to close the gaps in the individuals to obtain specimen-specific mitochondrial genomes. 

**Table 2:** Sequencing statistics.

set	reads	number of bases	mean read length	reads in mitochondrial contigs	assembly	no of contigs	Mean coverage
North Sea	37045	8146751	220	2135	18202	1	25
Baltic	11538	2832942	245	474	16312	4	7
Iceland	14483	3476793	240	684	17819	2	9

Some bivalves, namely Mytiloida, freshwater mussels of the superfamily Unionoidea, and marine clams of the order Veneroida exhibit so-called doubly uniparental inheritance (DUI). The species with DUI have two different kinds of mitochondrial genomes, a male (M) and female (F) one, between which differences in gene order and sequence divergence of up to 20 % can occur [20,21]. While females have only the F genome, males are heteroplasmic and possess F and M genomes, which are differentially expressed in somatic tissues (F) and in gonads (M) [22]. Among the three complete mitochondrial genomes we report in this study we did not find comparable levels of sequence heterogeneity or gene rearrangements known from species with DUI. Further sequencing of selected regions of 17 specimens (see below) did not recover highly heterogeneous sequences either, indicating either lack of DUI in this species, sampling of only female specimens, or only slight heterogeneity between F and M type mitochondrial genomes. The data of this study thus are not sufficient to determine the presence or absence of DUI in this species. 

The coding capacity of the mt genome is alike that of all other Metazoan mitochondria. As was observed in other Bivalvia, We could not find the very short atp8 gene. Presumably its similarity to known atp8 genes has degraded to levels below detectability. 

#### tRNAs

TRNAscan [23] with default settings did not detect more than 7 tRNA genes in the mitochondrial genome. Using the nematode specific parameters we were able to detect additional, but not a complete set of tRNAs. We thus used ARWEN [24] to predict mitochondrial tRNA genes. Most of the common tRNAs were detected by this tool but the tRNA for Glutamine was missing. A recent study showed that tRNA detection in Metazoa can be improved [25]. Using the software described there we were able to detect the tRNA for Glutamine (Table S1). Thus, the *A. islandica* mitochondrial genome contains tRNA genes for each amino acid. We detected two tRNA Glu genes, which is uncommon in Metazoa. They have a significantly different GC content (tRNA-Glu1 at position 8 to 81 16.2 versus 37.9 for tRNA-Glu2 at position 15409 to 15474) and sequence. However, duplication and rapid decay of tRNA genes was previously observed in a bivalve [26]. The second tRNA Glu might be a rare example of a not yet vanished gene duplication. We detected only a tRNA Ser recognizing UCN but failed to find the commonly found tRNA-Ser recognizing AGY despite the fact that these codons are used in the mt genome. We then searched for potential tRNA-like folds using Mfold [27] in intergenic regions but were not successful. It might therefore be possible that this tRNA is imported into the mitochondrion. We can, however, not exclude that a rather uncommon tRNA structure escaped our search. 

#### Gene order and synteny

**Figure 1:**
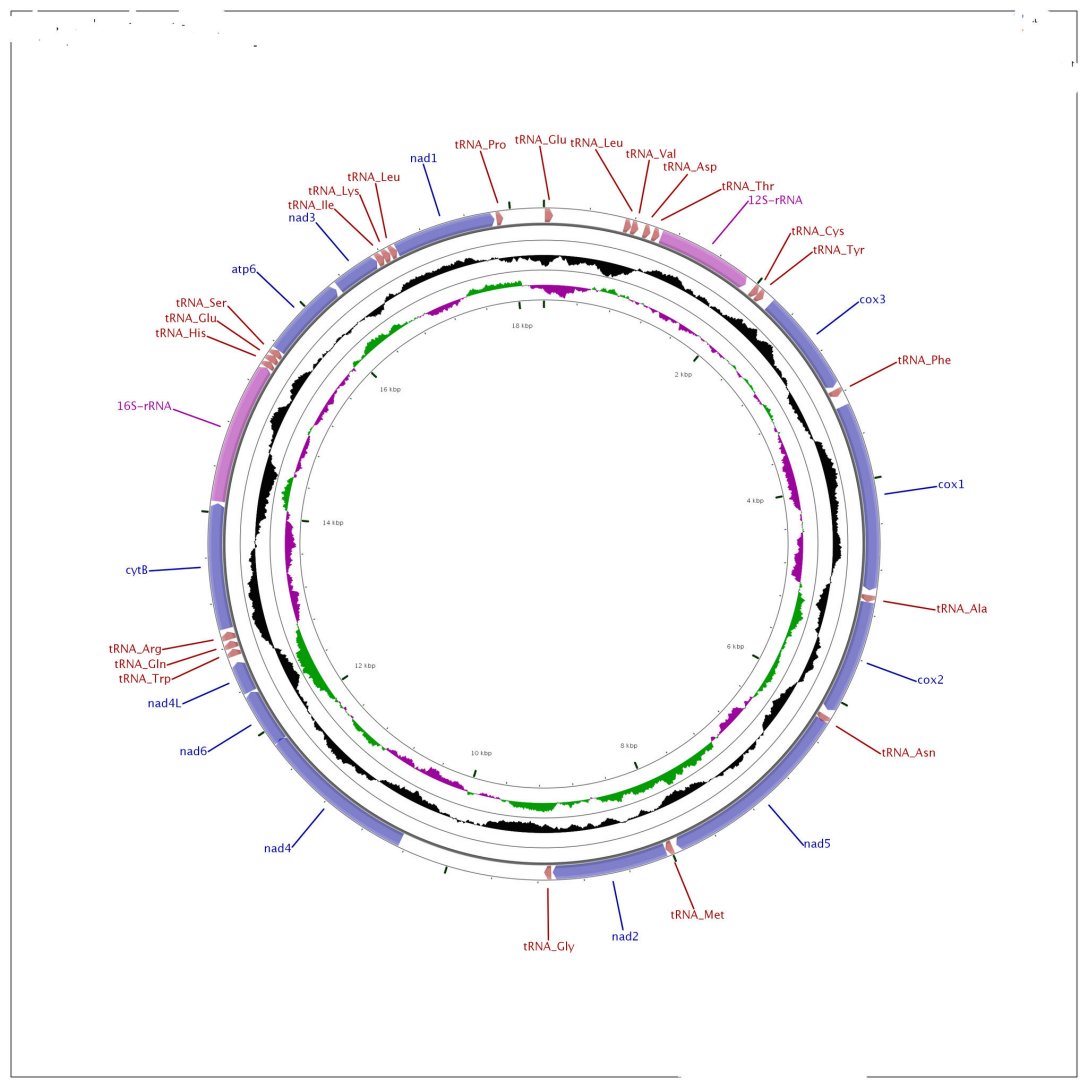
Map of the mitochondrial genome of *A. islandica*. The image was prepared using the CGView Server at http://stothard.afns.ualberta.ca/cgi-bin/cgview_server/. The Outer circle shows the genes, the middle circle the GC content and the inner circle the GC skew.

Most bivalve mitochondria sequenced to date have all their genes on only one strand. Only a few bivalves distribute their mitochondrial genes between DNA strands, e.g. *Lampsilis ornata* from the Unionida [28]. *A. islandica* encodes all genes on the same strand with the order of genes shown in Figure 1. This specific order is not completely shared by any other currently known mitochondrial genome of Bivalvia. Gene order, or synteny, can be retained in closely related species and therefore can be utilized as a marker of relatedness in the same sense as sequence similarity. Indeed, within the superfamilies of the order Heterodonta the gene order is highly conserved, only a few synteny breaks occur (Table S5: Gene order). 

Close inspection of the *A. islandica* gene order revealed that some genes share the same neighbors as in other bivalve species of the order Veneroida. The genes cytB, l-RNA, atp6, and nad3 occur in the same order as in *A. islandica* in all *Paphia* species of the family Veneridae. They differ, however, in that there is an insertion of a tRNA (tRNA_His) between l-RNA and atp6 in the *A. islandica* mitochondrial genome. Furthermore, *Meretrix* species and *Venerupis* have a similar, albeit more diversified gene cluster, only nad4 and three tRNAs are inserted between l-RNA and atp6. Thus, the tight clustering of these genes could be ancestral to these groups, whereas the location of other genes is not conserved. 

### Phylogeny

**Figure 2:**
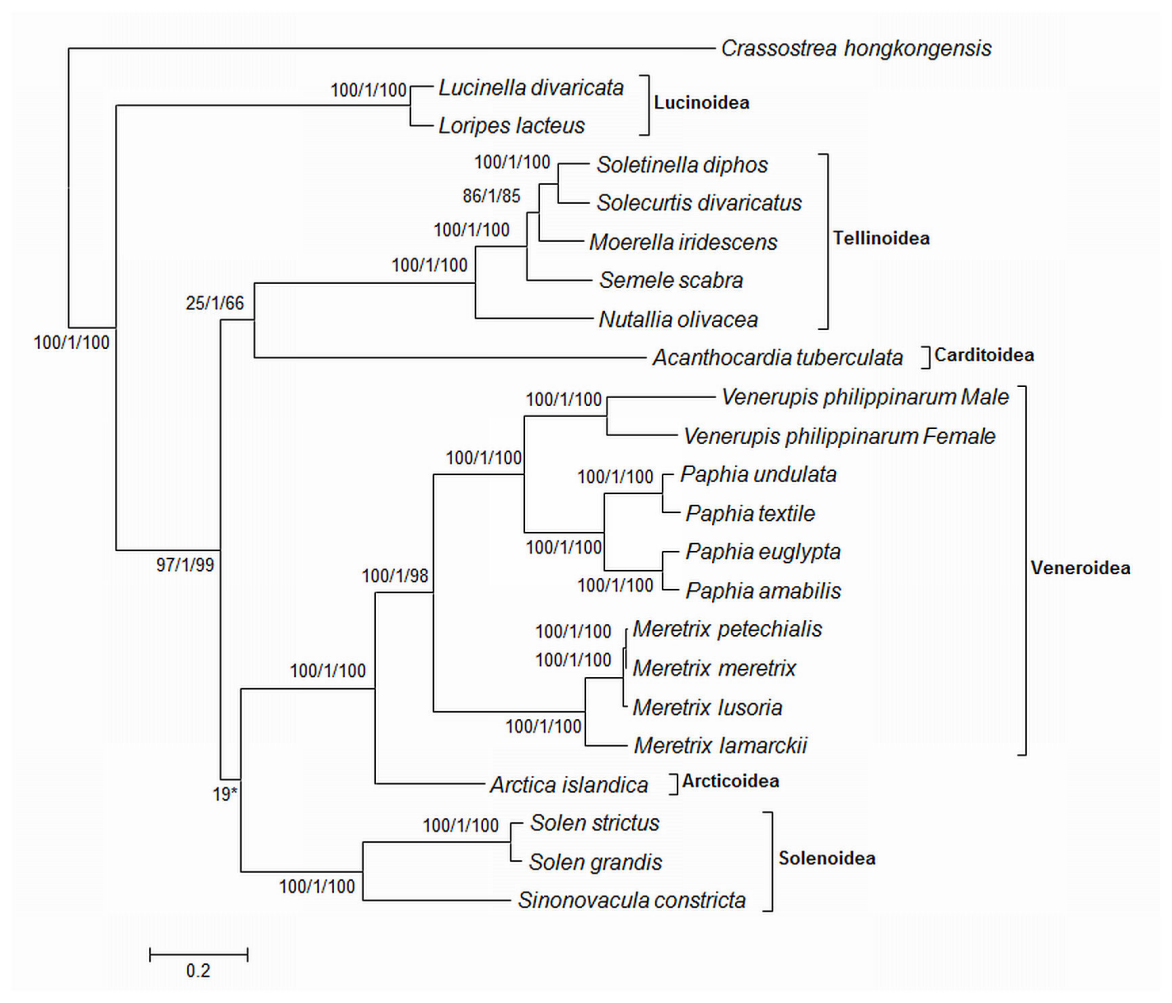
Molecular phylogeny of the bivalve subclass Heterodonta using concatenated protein sets from complete mitochondrial genomes. The evolutionary history was inferred using the Neighbor-Joining (NJ) method [37], MrBayes, and Maximum likelihood (ML) analysis. The tree shows the topology of the ML tree and the values next to the branches indicate (ordered from left to right) Percentage support by ML tree/Posterior probability of MrBayes tree/) Percentage support by NJ tree. The tree was rooted using *Crassostrea hongkongensis* as an outgroup. The tree is drawn to scale, with branch lengths measured in the number of substitutions per site. Evolutionary analyses were conducted in MEGA5 [38] (NJ and ML) and with MrBayes [39]. The asterisk indicates that the Solenoida branch is associated with Tellinoidea in NJ and MrBayes analyses.

Mitochondrial sequences are widely used to infer phylogenetic relationships among organisms. So far, 20 species within the bivalve subclass Heterodonta have their mitochondrial genomes sequenced. We took advantage of this data resource, concatenated the translated protein sequences of each species, and aligned them using muscle [29]. The phylogenetic relationships within the Heterodonta were reconstructed based on this concatenated alignment using maximum likelihood (ML), Neighbor Joining (NJ), and Bayes based methods and rooted with *Crassostrea* (Bivalvia, Pteriomorphia; Figure 2). All species cluster in their respective families. We noted that the position of *Sinonovacula constricta* in the taxonomy provided by NCBI is different from its position in our phylogenetic reconstruction yet agrees with a previous study [30]. According to the NJ, ML, and MrBayes phylogeny, *A. islandica* branches at the basis of Veneroidea and is the sister group to *Paphia*, *Meretrix*, and *Venerupis*. This branching order is in good agreement with the observed syntenic regions shared between Veneroidea and *A. islandica*, further emphasizing the relationship between these families. 

### Polymorphisms

**Figure 3:**
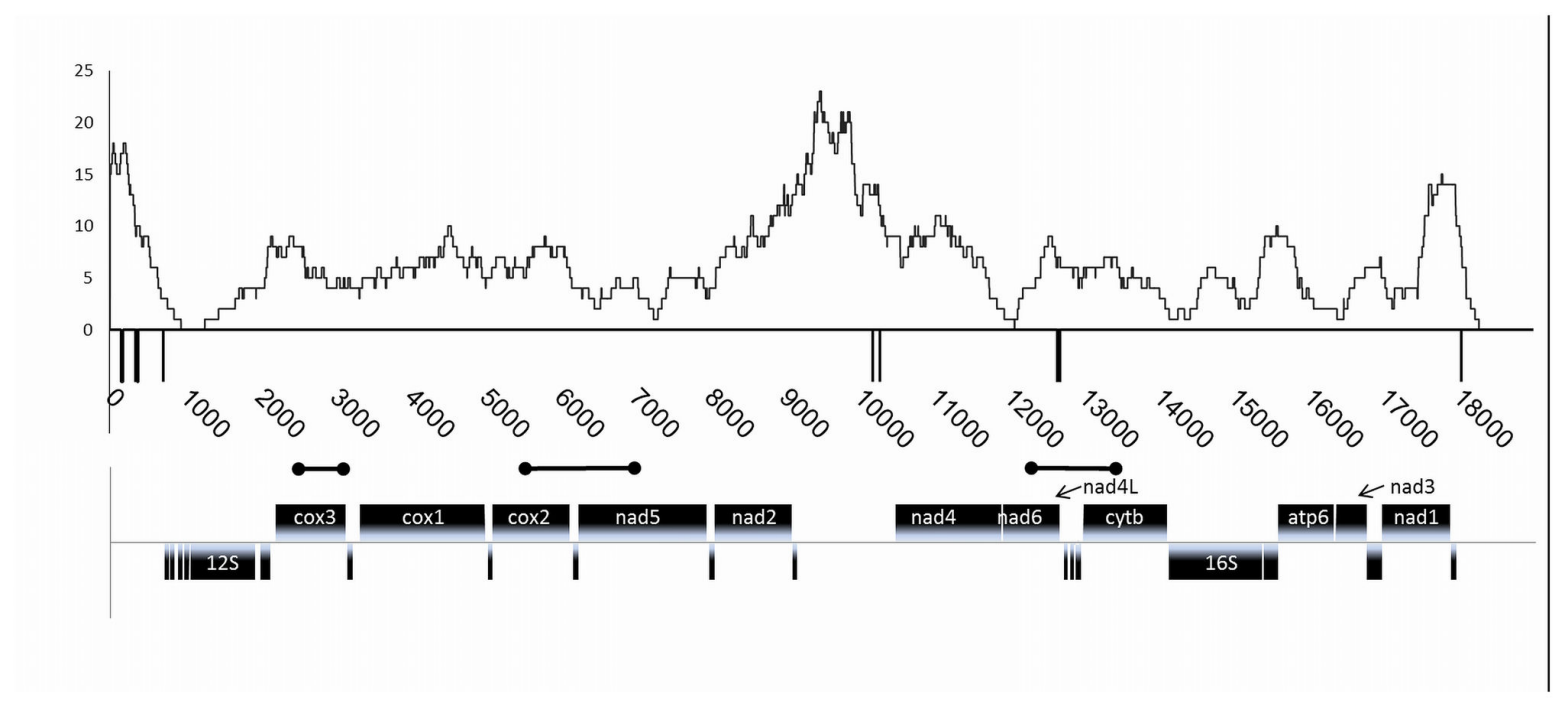
Polymorphisms in the mitochondrial genome. Single nucleotide polymorphisms (SNPs) were counted in a sliding window of 500 with a step size of 1. All genes are depicted below the SNP frequency curve. The indel regions are indicated by vertical lines below the sliding window curve. Protein coding genes (CDS) are shown above the second line and RNA species below that same line as boxes. The CDS and the 12S and 16S RNA genes are named, while the tRNA genes are only depicted as rectangles. The bar-bell shapes indicate the amplified regions from 17 specimen.

We sequenced the entire mitochondrial genomes of one specimen of *A. islandica* from each of the three sampling sites. This gave us the opportunity to assess the distribution of polymorphisms along the mitochondrial genome. All polymorphisms detected are relatively evenly distributed over the whole genome (Table S5: polymorphisms). The 12S rRNA region is an exception where over nearly 1 kb no polymorphism could be observed (Figure 3). Furthermore, the 16S rRNA region is also comparably free of polymorphisms, indicating higher evolutionary constraints for the rRNA genes compared to protein coding genes. The two regions devoid of genes are more prone to accumulate polymorphisms than the genic regions. This is even more emphasized by the fact that most indels (4 of 5 observed) are located in these regions. 

The Baltic Sea specimen exhibits the highest number of SNPs compared to the other two, followed by the North Sea specimen (Table 3). Later examination of further specimens showed that the Baltic and North Sea mt genomes represent the two common haplotypes, whereas the Iceland MT genome is, at the examined positions, a mixture between these two haplotypes with the majority of SNPs shared with the North Sea haplotype. 

**Table 3:** Polymorphisms in the completed MT genomes of *A. islandica*.

	differences to consensus	Variants in consensus	Total unique SNPs
Baltic	167	30	197
North Sea	40	12	52
Iceland	21	2	23

We counted transitions and transversions and calculated the proportion of these to be 9 to 1. 2.7 % of intergenic and 1.18 % of genic sites were polymorphic, respectively, with 159 coding SNPs in total (Table S2). Examining the coding SNPs revealed that the proportion of non-synonymous to synonymous SNPs (dN/dS) is 0.25 (Table S3). We searched for potential open reading frames in the regions free of canonical genes and found one at position 9196 to 9855 with very slight similarity to the nad2 gene. This ORF might be a remnant of a gene duplication. The analysis of synonymous and non-synonymous SNPs of each gene showed that this ORF has accumulated significant amounts of non-synonymous polymorphisms indicating a decaying gene (Table S4).

We wanted to obtain a better insight into SNP differences within populations by analyzing distinct parts of the mt genome in more detail in several specimen. To avoid a selection bias we chose regions relatively evenly distributed over the mt genome, but containing polymorphisms according to the complete genome sequences (Figure 3). The respective regions were amplified from a total of 17 specimens and subsequently sequenced using Sanger technology. We observed only variation employing two bases at each polymorphic position (SNPs). The results strongly suggest that only one mutational event per site led to the observed pattern. In total, we found 17 T/C, 12 A/G, and 2 G/C polymorphisms, respectively. Furthermore, two single base-pair indels contribute to the sequence variability in these regions. We also observed some individual polymorphisms not shared with other specimens potentially representing individual point mutations or markers for population substructures. In the North Sea population only one of these polymorphisms was detected, whereas the Baltic Sea and Icelandic samples contained 10 and 21, respectively (data not shown). 

**Figure 4:**
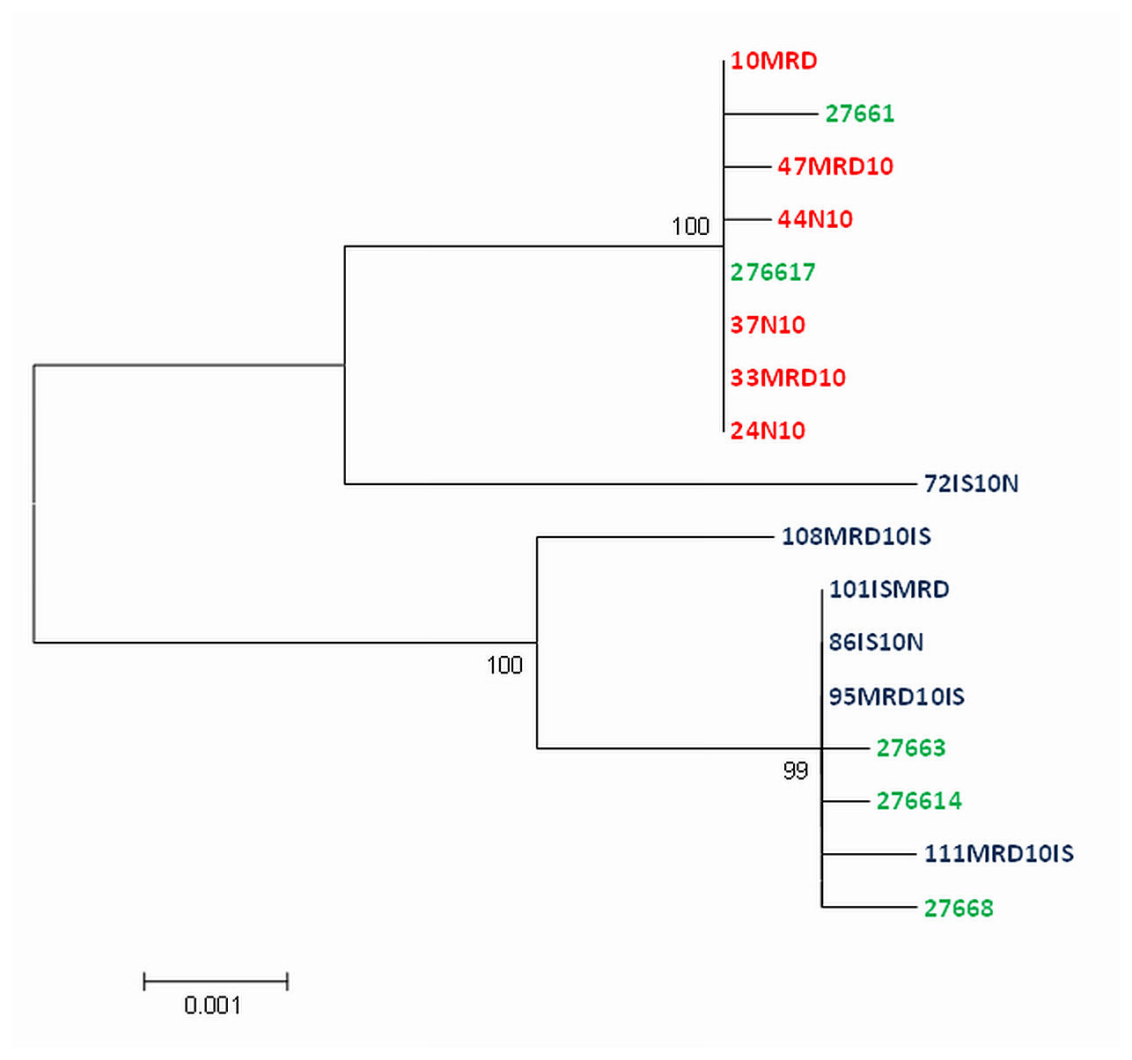
Molecular Phylogenetic analysis of the concatenated nucleotide sequences from three regions of the mitochondrial genome (2470 to 3050; 5490 to 6890; 12230 to 13380) using the Maximum Likelihood method based on the Hasegawa-Kishino-Yano model [40]. Initial tree(s) for the heuristic search were obtained automatically by applying Neighbor-Join and BioNJ algorithms to a matrix of pairwise distances estimated using the Maximum Composite Likelihood (MCL) approach, and then selecting the topology with superior log likelihood value. The tree is drawn to scale, with branch lengths measured in the number of substitutions per site. Evolutionary analyses were conducted in MEGA5 [38]. Blue: Iceland; Green: Baltic Sea; Red: North Sea.

Investigating the DNA sequences in all three genomic regions in their entirety, we found two distinct groups of haplotypes (Table S5: SNPs in amplified regions). The two major haplotypes are present both in the Baltic Sea population (4 specimens with major haplotype 1 and 2 specimens with major haplotype 2, respectively), whereas only one of these haplotypes could be found in each of the other two populations. The North Sea Population exhibited the major haplotype 1 only, whereas the Iceland population showed major haplotype 2 in four out of six specimens. The remaining two Icelandic specimens (108MRD10IS and 72IS10N) had different haplotypes. The haplotype of 72IS10N differs from one of the major haplotypes in the third region by only one polymorphism, whereas we observed 3 differences in region 1 (out of seven sites) and 2 (out of 13 sites), respectively. The haplotype of 108MRD10IS has one difference to the other major haplotype in region 2, but appears to have inserted a portion from a different haplotype in the third region. Since it is unlikely that independent mutations led to these SNPs, this strongly suggests a potential partial mixture of haplotypes. We also performed a phylogenetic analysis of the concatenated amplicons which gave an alignment of 3025 positions or 16.5 % of the whole genome. A model test performed in MEGA 5 defined the Hasegava-Kishino-Jano model as best fitting model. Using this model the maximum likelihood calculation gave a tree with two clusters (Figure 4). The two sequences from 72IS10N and 108MRD10IS were clearly separated from these clusters as would be expected if a mixed ancestry is assumed. We also performed statistical tests using available software tools (PhiPack[31]; RDP [32]) but found no statistically significant recombination breakpoint, potentially due to the small number of examinable SNPs.

Such apparently recombined mitochondrial haplotypes can only occur if heteroplasmy, i.e. presence of more than one type of MT genome in an individual, exists at least in some life stages [33]. Whether these mt genomes represent only slightly different F and M genomes or are variants which can just co-exist in the mitochondrion of *A. islandica* remains to be shown by further analysis of additional specimens. 

The predominant Iceland haplotype is missing in the North Sea sampling site, which could be due to the relatively small number of sampled specimens, which are possibly closely related. Alternatively, the presence of only one haplotype at the North Sea sampling site could also be due to the increased environmental pressure from the combined effect of fisheries, eutrophication and increasing water temperatures that compromise recruitment in this population [6] leading to a population bottleneck. This is further underlined by the fact that this population has also nearly no individual variation. On the other hand the Baltic Sea population exhibits both common haplotypes and a moderate number of individual polymorphisms. Taken together, the Icelandic population has the most variable mt genomes, representing a resource for diversity whereas the more southward marginal populations are marked by more homogenous mt genomes, especially in the strongly impacted German Bight. 

## Conclusion

We here presented the whole mitochondrial genome sequence of *Arctica islandica*, a species with an outstanding potential for longevity research. The MT genome encodes only one tRNA-Ser, a feature very uncommon among most Metazoa and here firstly described for a bivalve. Polymorphisms are less frequent in structural RNAs than coding genes and accumulate preferentially in intergenic regions where also remnants of gene duplications can reside. The analysis of selected regions revealed potential haplotype mixing. The variation of haplotypes in the MT genomes could be used as good measure for population diversity and thus for assessment of the habitat quality.

## Materials and Methods

### Sampling

All sampling sites are not specially protected areas of the marine environment and fishing is allowed. *A. islandica* is not an endangered species and thus no regulations on their sampling currently apply. Sampling sites were: Baltic Sea, Kiel Bay: N 54°32.6´- E 10°42.1´; Iceland: N 66° 01`44 - W 14° 50`91; North Sea, Helgoland: N 54°10`44- E 007° 48`12. 

### DNA isolation and sequencing

For the determination of the mitochondrial genome sequence DNA was extracted from specimen using the Qiagen DNeasy Blood & Tissue Kit. The genomic library for the 454 flx sequencing machine was prepared according to the manufacturers protocols (Roche). The raw data from the sequencing runs were searched for mitochondria like sequences with NC_003354 (Venerupis philippinarum) using BLAST. The raw reads then were assembled using Newbler software with default values. The reads not placed into mitochondrial contigs were checked for presence of sequences indicating nuclear copies of the mt genome (NUMTs). In no case we detected fusions of mt genomic sequences to likely genomic sequences, which is likely due to the much lower coverage of genomic sequences with raw reads. We also analysed the raw reads for potential presence of an alternative mt genome indicated by sequence differences within species but found no hint for heteroplasmy in the completely sequenced specimen. Initial assembly of the North Sea specimens yielded one contig covering nearly the whole genome. The circle was closed by sequencing a PCR product resulting from amplification of mitochondrial DNA using custom primers adjacent to the remaining gap. Gaps in the assembly for specimens from the Baltic Sea and Iceland were closed using the North Sea sequence as a guide for orientation of the individual contigs yielding finally three high quality genomes. The three consensus sequences were aligned and manually curated within the STADEN package [34] and the sequence differences between the three mt genomes were extracted using custom made software tools. Subsequently, selected regions of the genome were amplified from each specimen. The resulting sequences were aligned to the combined reference sequence and the polymorphic sites counted.

### Phylogeny reconstruction

The protein coding genes of the mitochondrial genomes of Veneroida species were retrieved from GenBank and aligned to their counterparts using muscle [29]. These alignments were concatenated with Scafos [35] There were a total of 3658 positions in the final dataset. The appropriate model of evolution for Maximum Likelihood calculation (Whelan And Goldman + Freq. model [36]) was selected by testing several models using the MEGA software. A discrete Gamma distribution was used to model evolutionary rate differences among sites (5 categories (+*G*, parameter = 1.9354)). The The MrBayes model was a variable GTR model, and the NJ analysis used the JTT model. The trees were rooted using *Crassostrea hongkongensis* as an outgroup. 

## Supporting Information

Table S1 **Comparison of software tools for the detection of tRNAs.**
(DOCX)

Table S2 **Detected polymorphisms between the three sequenced genomes.**
(DOCX)

Table S3 **The number of synonymous versus non-synonymous exchanges in coding portions of the mitochondrial genome.**
(DOCX)

Table S4 **The SNPs in genes of the mt genome.**
(DOCX)

Table S5 **Positions and kind of all detected polymorphisms; Polymorphisms in amplified regions; Synteny between Veneroida mitochondrial genomes.**
(XLSX)
