# Supplementary material for: The Mitochondrial Genome of Arctica islandica; Phylogeny and Variation
Source: PLoS One. 2013 Dec 2;8(12):e82857. doi: 10.1371/journal.pone.0082857 (PMC3847043; doi:10.1371/journal.pone.0082857)
Supplement: Table S1 — Comparison of software tools for the detection of tRNAs. (DOCX) [file pone.0082857.s001.docx]

Table S1: Comparison of software tools for the detection of tRNAs

| start | end | tRNA | ARWEN | tRNA_Scan-SE | MitFI |
| --- | --- | --- | --- | --- | --- |
| 8 | 81 | mtRNA-Glu | + | + | - |
| 699 | 761 | mtRNA-Leu | + | + | + |
| 765 | 827 | mtRNA-Val | + | + | + |
| 878 | 940 | mtRNA-Asp | + | + | + |
| 963 | 1025 | mtRNA-Thr | + | + | + |
| 1976 | 2038 | mtRNA-Cys | + | + | + |
| 2039 | 2100 | mtRNA-Tyr | + | - | + |
| 3136 | 3199 | mtRNA-Phe | + | + | + |
| 3272 | 3336 | mtRNA-Phe | - | - | + |
| 5008 | 5071 | mtRNA-Ala | + | + | + |
| 6145 | 6208 | mtRNA-Asn | + | + | + |
| 7960 | 8025 | mtRNA-Met | + | - | + |
| 9063 | 9126 | mtRNA-Gly | + | + | + |
| 12687 | 12751 | mtRNA-Trp | + | + | + |
| 12760 | 12826 | mtRNA-Gln | - | - | + |
| 12838 | 12900 | mtRNA-Arg | + | - | + |
| 15349 | 15408 | mtRNA-His | + | + | + |
| 15409 | 15474 | mtRNA-Glu | + | + | + |
| 15471 | 15533 | mtRNA-Ser | + | - | + |
| 16722 | 16787 | mtRNA-Ile | + | + | + |
| 16788 | 16853 | mtRNA-Lys | + | - | + |
| 16855 | 16918 | mtRNA-Leu | + | + | + |
| 17847 | 17908 | mtRNA-Pro | + | + | + |
