# Supplementary material for: The Mitochondrial Genome of Arctica islandica; Phylogeny and Variation
Source: PLoS One. 2013 Dec 2;8(12):e82857. doi: 10.1371/journal.pone.0082857 (PMC3847043; doi:10.1371/journal.pone.0082857)
Supplement: Table S2 — Detected polymorphisms between the three sequenced genomes. (DOCX) [file pone.0082857.s002.docx]

Table S2: Detected polymorphisms between the three sequenced genomes.

| kind of SNP |  |  | tRNA | rRNA | intergenic | coding |
| --- | --- | --- | --- | --- | --- | --- |
| A>T | 4 | transversion | 0 | 0 | 4 | 0 |
| T>A | 7 | transversion | 0 | 0 | 4 | 3 |
| G>C | 1 | transversion | 0 | 0 | 0 | 1 |
| C>G | 7 | transversion | 1 | 0 | 2 | 4 |
| A>C | 1 | transversion | 0 | 0 | 1 | 0 |
| A>G | 57 | transition | 0 | 2 | 15 | 40 |
| T>C | 53 | transition | 1 | 2 | 14 | 36 |
| T>G | 2 | transversion | 1 | 0 | 1 | 0 |
| G>A | 59 | transition | 3 | 3 | 16 | 37 |
| G>T | 1 | transversion | 0 | 0 | 1 | 0 |
| C>A | 0 | transversion | 0 | 0 | 0 | 0 |
| C>T | 49 | transition | 1 | 2 | 8 | 38 |
| deletion | 30 |  | 0 | 0 | 30 | 0 |
| insertion | 1 |  | 0 | 0 | 1 | 0 |
|  |  |  | 7 | 9 | 97 | 159 |
