# Supplementary material for: The Mitochondrial Genome of Arctica islandica; Phylogeny and Variation
Source: PLoS One. 2013 Dec 2;8(12):e82857. doi: 10.1371/journal.pone.0082857 (PMC3847043; doi:10.1371/journal.pone.0082857)
Supplement: Table S3 — The number of synonymous versus non-synonymous exchanges in coding portions of the mitochondrial genome. (DOCX) [file pone.0082857.s003.docx]

Table S3: The number of synonymous versus non-synonymous exchanges in coding portions of the mitochondrial genome.

| genic SNPs |  | All | synonymous | non-synonymous | Proportion (dN/dS) |
| --- | --- | --- | --- | --- | --- |
|  | 1st position | 33 | 15 | 18 | 1.2 |
|  | 2nd position | 14 | 0 | 14 | na |
|  | 3rd position | 111 | 111 | 0 | 0 |
|  | All | 158 | 126 | 32 | 0.25 |

*1SNP is potentially a sequencing error and was not included

We found that 2.7 % of intergenic and 1.18 % of genic sites were polymorphic, respectively. The coding SNPs showed a ratio of 1:4 concerning non-synonymous to synonymous SNPs.
