# Supplementary material for: The Mitochondrial Genome of Arctica islandica; Phylogeny and Variation
Source: PLoS One. 2013 Dec 2;8(12):e82857. doi: 10.1371/journal.pone.0082857 (PMC3847043; doi:10.1371/journal.pone.0082857)
Supplement: Table S4 — The SNPs in genes of the mt genome. (DOCX) [file pone.0082857.s004.docx]

Table S4: The SNPs in genes of the mt genome

| gene | start | end | SNPs | N | S | indel |
| --- | --- | --- | --- | --- | --- | --- |
| cox3 | 2179 | 3108 | 12 | 1 | 11 |  |
| cox1 | 3295 | 4965 | 21 | 4 | 17 |  |
| cox2 | 5072 | 6094 | 13 | 5 | 8 |  |
| nad5 | 6195 | 7919 | 13 | 0 | 13 |  |
| nad2 | 8028 | 9050 | 14 | 4 | 10 |  |
| nad2 decaying | 9196 | 9855 | 20 | 14 | 6 |  |
| nad4 | 10436 | 11851 | 25 | 4 | 21 |  |
| nad6 | 11835 | 12338 | 2 | 0 | 2 |  |
| nad4L | 12344 | 12628 | 3 | 0 | 3 |  |
| cytb | 12940 | 14059 | 13 | 0 | 13 |  |
| atp6 | 15534 | 16277 | 10 | 0 | 10 |  |
| nad3 | 16307 | 16717 | 2 | 0 | 2 |  |
| nad1 | 16920 | 17831 | 10 | 0 | 10 |  |
| atp8 | nd | nd |  |  |  |  |
|  |  |  |  |  |  |  |
| rDNA-12S | 1039 | 1901 | 1 |  |  |  |
| rDNA-16S | 14077 | 15348 | 8 |  |  |  |
|  |  |  |  |  |  |  |
| mtRNA-Glu | 8 | 81 | 1 |  |  | 1 |
| mtRNA-Leu | 699 | 761 | 1 |  |  |  |
| mtRNA-Val | 765 | 827 | 0 |  |  |  |
| mtRNA-Asp | 878 | 940 | 1 |  |  |  |
| mtRNA-Thr | 963 | 1025 | 0 |  |  |  |
| mtRNA-Cys | 1976 | 2038 | 0 |  |  |  |
| mtRNA-Tyr | 2039 | 2100 | 0 |  |  |  |
| mtRNA-Phe | 3136 | 3199 | 1 |  |  |  |
| mtRNA-Phe | 3272 | 3336 | 0 |  |  |  |
| mtRNA-Ala | 5008 | 5071 | 0 |  |  |  |
| mtRNA-Asn | 6145 | 6208 | 0 |  |  |  |
| mtRNA-Met | 7960 | 8025 | 0 |  |  |  |
| mtRNA-Gly | 9063 | 9126 | 0 |  |  |  |
| mtRNA-Trp | 12687 | 12751 | 0 |  |  |  |
| mtRNA-Gln | 12760 | 12826 | 0 |  |  |  |
| mtRNA-Arg | 12838 | 12900 | 2 |  |  |  |
| mtRNA-His | 15349 | 15408 | 0 |  |  |  |
| mtRNA-Glu | 15409 | 15474 | 0 |  |  |  |
| mtRNA-Ser | 15471 | 15533 | 1 |  |  |  |
| mtRNA-Ile | 16722 | 16787 | 0 |  |  |  |
| mtRNA-Lys | 16788 | 16853 | 0 |  |  |  |
| mtRNA-Leu | 16855 | 16918 | 0 |  |  |  |
| mtRNA-Pro | 17847 | 17908 | 0 |  |  |  |

N=non-synonymous polymorphism; S=synonymous polymorphism
